# Algorithm for the Early Diagnosis and Treatment of Patients with Cross Reactive Immunologic Material-Negative Classic Infantile Pompe Disease: A Step towards Improving the Efficacy of ERT

**DOI:** 10.1371/journal.pone.0067052

**Published:** 2013-06-25

**Authors:** Suhrad G. Banugaria, Sean N. Prater, Trusha T. Patel, Stephanie M. DeArmey, Christie Milleson, Kathryn B. Sheets, Deeksha S. Bali, Catherine W. Rehder, Julian A. J. Raiman, Raymond A. Wang, Francois Labarthe, Joel Charrow, Paul Harmatz, Pranesh Chakraborty, Amy S. Rosenberg, Priya S. Kishnani

**Affiliations:** 1 Division of Medical Genetics, Department of Pediatrics, Duke University Medical Center, Durham, North Carolina, United States of America; 2 Clinical Molecular Diagnostic Laboratories, Duke University Health System, Durham, North Carolina, United States of America; 3 Division of Clinical and Metabolic Genetics, The Hospital for Sick Children, University of Toronto, Toronto, ON, Canada; 4 Children’s Hospital of Orange County, Orange, California, United States of America; 5 Hôpital Clocheville, University Hospital, Tours, France; 6 Department of Pediatrics, Feinberg School of Medicine, Northwestern University, Chicago, Illinois, United States of America; 7 Children's Hospital and Research Center Oakland, Oakland, California, United States of America; 8 Department of Pediatrics, University of Ottawa, Ottawa, Canada; 9 Division of Therapeutic Proteins, Office of Biotechnology Products, Center for Drug Evaluation and Research, United States Food and Drug Administration, Bethesda, Maryland, United States of America; University Hospital S. Maria della Misericordia, Udine, Italy

## Abstract

**Objective:**

Although enzyme replacement therapy (ERT) is a highly effective therapy, CRIM-negative (CN) infantile Pompe disease (IPD) patients typically mount a strong immune response which abrogates the efficacy of ERT, resulting in clinical decline and death. This study was designed to demonstrate that immune tolerance induction (ITI) prevents or diminishes the development of antibody titers, resulting in a better clinical outcome compared to CN IPD patients treated with ERT monotherapy.

**Methods:**

We evaluated the safety, efficacy and feasibility of a clinical algorithm designed to accurately identify CN IPD patients and minimize delays between CRIM status determination and initiation of an ITI regimen (combination of rituximab, methotrexate and IVIG) concurrent with ERT. Clinical and laboratory data including measures of efficacy analysis for response to ERT were analyzed and compared to CN IPD patients treated with ERT monotherapy.

**Results:**

Seven CN IPD patients were identified and started on the ITI regimen concurrent with ERT. Median time from diagnosis of CN status to commencement of ERT and ITI was 0.5 months (range: 0.1–1.6 months). At baseline, all patients had significant cardiomyopathy and all but one required respiratory support. The ITI regimen was safely tolerated in all seven cases. Four patients never seroconverted and remained antibody-free. One patient died from respiratory failure. Two patients required another course of the ITI regimen. In addition to their clinical improvement, the antibody titers observed in these patients were much lower than those seen in ERT monotherapy treated CN patients.

**Conclusions:**

The ITI regimen appears safe and efficacious and holds promise in altering the natural history of CN IPD by increasing ERT efficacy. An algorithm such as this substantiates the benefits of accelerated diagnosis and management of CN IPD patients, thus, further supporting the importance of early identification and treatment initiation with newborn screening for IPD.

## Introduction

Pompe disease (OMIM 232300; acid maltase deficiency, glycogen storage disease type II) is an autosomal recessive deficiency of lysosomal acid alpha-glucosidase (GAA; OMIM 606800) that results in progressive glycogen accumulation [1]. Classic infantile Pompe disease (IPD) is characterized by cardiomegaly, respiratory insufficiency, and profound hypotonia. Without treatment, death secondary to cardiorespiratory failure occurs prior to two years of age [2]. Enzyme replacement therapy (ERT) with recombinant human acid alpha glucosidase (rhGAA; alglucosidase alfa) has been commercially available since 2006, and has led to improved clinical outcome measures, including prolonged overall and ventilator-free survival in IPD patients [3]–[6]. While such improvements have been noted initially for the IPD population as a whole, marked variability and long-term unpredictability in treatment response remains a challenge. A host of endogenous and exogenous factors are believed to account for this, but have yet to be completely elucidated. Given the rapid disease progression, early diagnosis and treatment are critical, as even slight delays can result in a significantly altered clinical course [6], [7].

Despite some gaps in knowledge, certain factors have been identified as having prognostic value in IPD, most prominent among them being cross-reactive immunologic material (CRIM) status. CRIM-negative (CN) patients with two deleterious mutations and no GAA protein expression experience an initial response to ERT before entering a phase of devastating clinical decline at rate that approximates that observed in untreated IPD [8]. This clinical decline in CN cases is largely due to the development of high sustained anti-rhGAA antibody titers (HSAT). While there are some exceptions in which CRIM-positive (CP) patients develop HSAT and experience clinical decline similar to CN patients, the majority of CP patients with missense mutations and some residual GAA protein either do not mount an immune response or mount a transient low titer response, and exhibit a more favorable response to ERT monotherapy [8], [9].

Evidence from long-term clinical experience with four CN IPD patients has demonstrated successful immune tolerance induction (ITI) with a regimen of rituximab (RTX) and methotrexate (MTX) ± intravenous immunoglobulin (IVIG) in the treatment-naïve (n = 2) or early ERT (n = 2) setting [10], [11]. Patients in whom anti-rhGAA antibody titers were essentially eliminated showed greatly improved clinical response to ERT, thus demonstrating the great clinical utility of such immunomodulatory protocols in the management of IPD [10]. However, a significant difference between the naïve patients and those already receiving ERT was the amount of immune modulation needed: patients already receiving ERT prior to the initiation of immune modulation required prolonged immune modulation [10]. In another two CRIM-negative cases with an entrenched immune response, immune suppression was unsuccessful despite multiple attempts over several years with different agents [12], [13]. Although clinical experience and current literature on the use of ITI protocols are greatly limited, success is more likely when immune modulation is started at the onset of ERT (ERT-naïve setting) [14]. Yet, there is no established algorithm which clearly delineates the most efficient pathway for treatment once a diagnosis of CN IPD is made. Here, we describe an algorithm for rapid diagnosis and management of CN IPD, and demonstrate successful ITI with a regimen of rituximab, methotrexate ± intravenous immunoglobulin in the ERT-naïve setting. We also evaluate the effectiveness of this algorithm by examining clinical outcomes seen in the CN patients treated with ERT+ITI versus CN patients treated with ERT monotherapy.

## Patients and Methods

### Patient Identification and Algorithm for Rapid CRIM Status Determination and ITI Treatment

**Figure 1:**
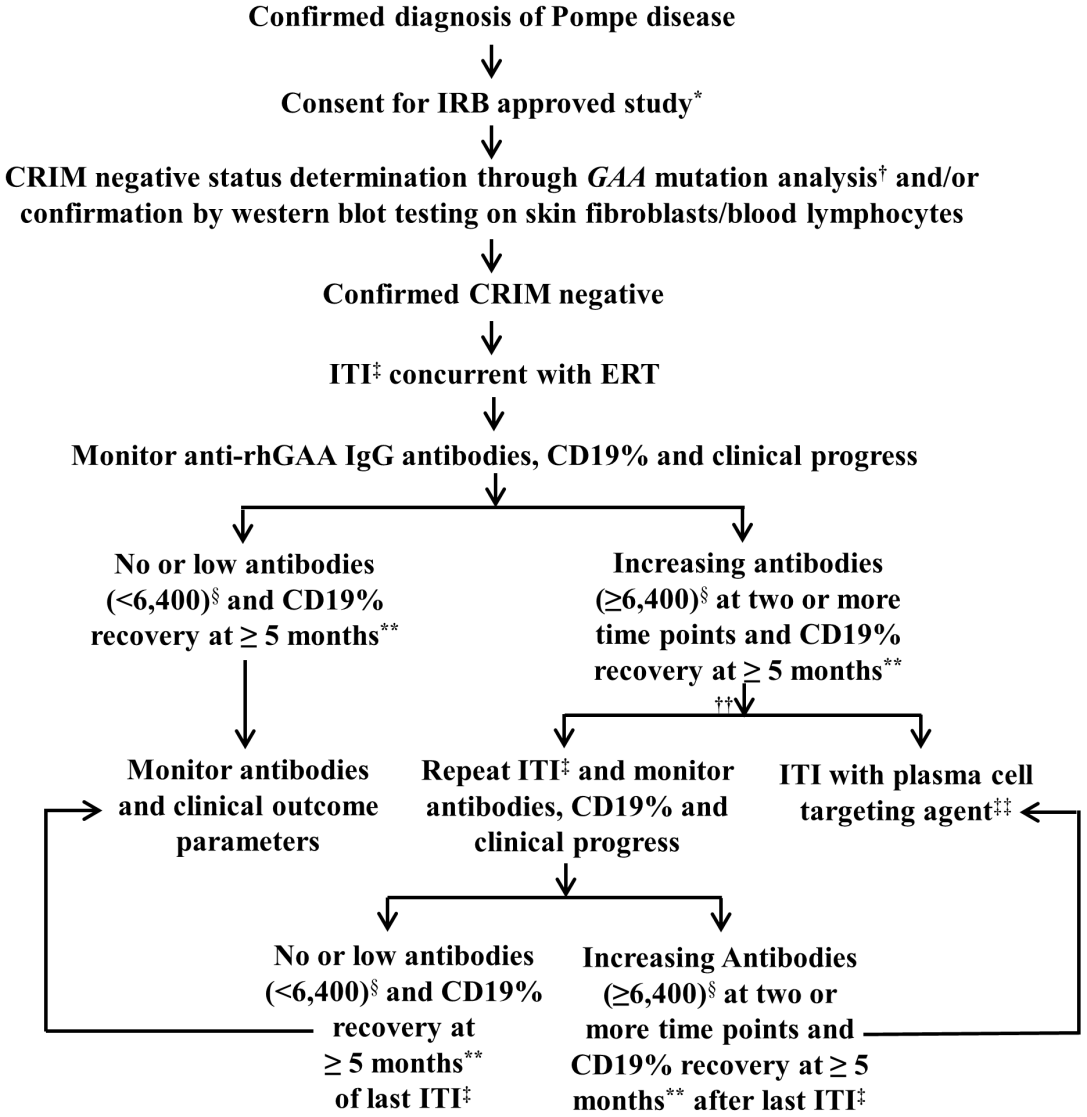
An algorithm for the management of cross-reactive immunologic material (CRIM)-negative (CN) infantile Pompe disease patients. ^*^Institutional review board (IRB) approved study (NCT01665326; www.clinicaltrials.gov) for rapid determination of CRIM status and long-term follow-up of response to treatment and ITI in Pompe disease. ^†^CN status determination from an established CRIM negative mutation database, which allows prediction of CN status in more than 90% cases [15]. ^‡^ITI regimen is shown in Figure 2. ^§^Based on the literature antibody titers sustained at ≥6,400 results in a suboptimal therapeutic response to ERT. For that reason, 6,400 was used a cutoff for further intervention [9], [19]. **Based on the half-life of rituximab, CD19% recovery is typically noted around 5 months. ^††^The decision to repeat the same ITI regimen (figure 3) or to administer ITI with a plasma-cell-targeting agent [20] should be based on multiple factors including, but not limited to, patients clinical status, CD19% and Fc_γ_ receptor polymorphism. ^‡‡^ITI regimen with plasma cell targeting agent such as bortezomib has been described previously [20].

**Figure 2:**
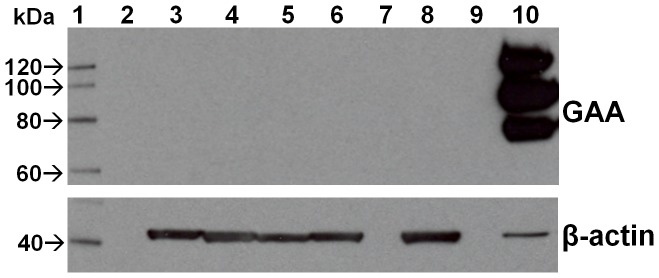
Representative Western gel blot showing CRIM negative status of four patients (lanes 3–6). Lane 1- protein magic marker; lane 8 -CRIM negative control cell line; lane 10 - normal human fibroblast (NHF) control; Lanes 2, 7 and 9 - left empty. 20 ug of skin fibroblast cell protein extract was loaded for each patient lane and 2.5 ug protein was loaded for NHF. Western blot was probed with anti-GAA antibody and ß-Actin was used as a protein loading control.

As part of our existing Duke Institutional Review Board (IRB)-approved study (Pro00001562; NCT01665326, www.clinicaltrials.gov), written informed consent was obtained by telephone from the patients’ parent(s) or legal guardian(s) for determination of CRIM status and long-term follow-up of each patient. The patient’s local physician acted as a third party witness to the telephone consent. The consent form was signed by the parent(s)/legal guardian(s) and returned via email or fax to the Duke study staff for his/her signature. A copy of the fully signed consent form was returned to the parent(s)/legal guardian(s). An algorithm for the rapid diagnosis and timely initiation of ITI was developed and implemented specifically for CN IPD patients (Figure 1). As per the algorithm, upon diagnostic confirmation of IPD, CN status was rapidly inferred by mutation analysis, using an established mutation database, which has allowed prediction of CN status in more than 90% cases [15] CRIM negative status was further confirmed using western blot analysis on skin fibroblast cell extracts, if none of the GAA protein processing forms (unprocessed precursor band at 110 kDa or processed forms bands at 95, 76 and 70 kDa) were detected (Figure 2) [8].

**Figure 3:**
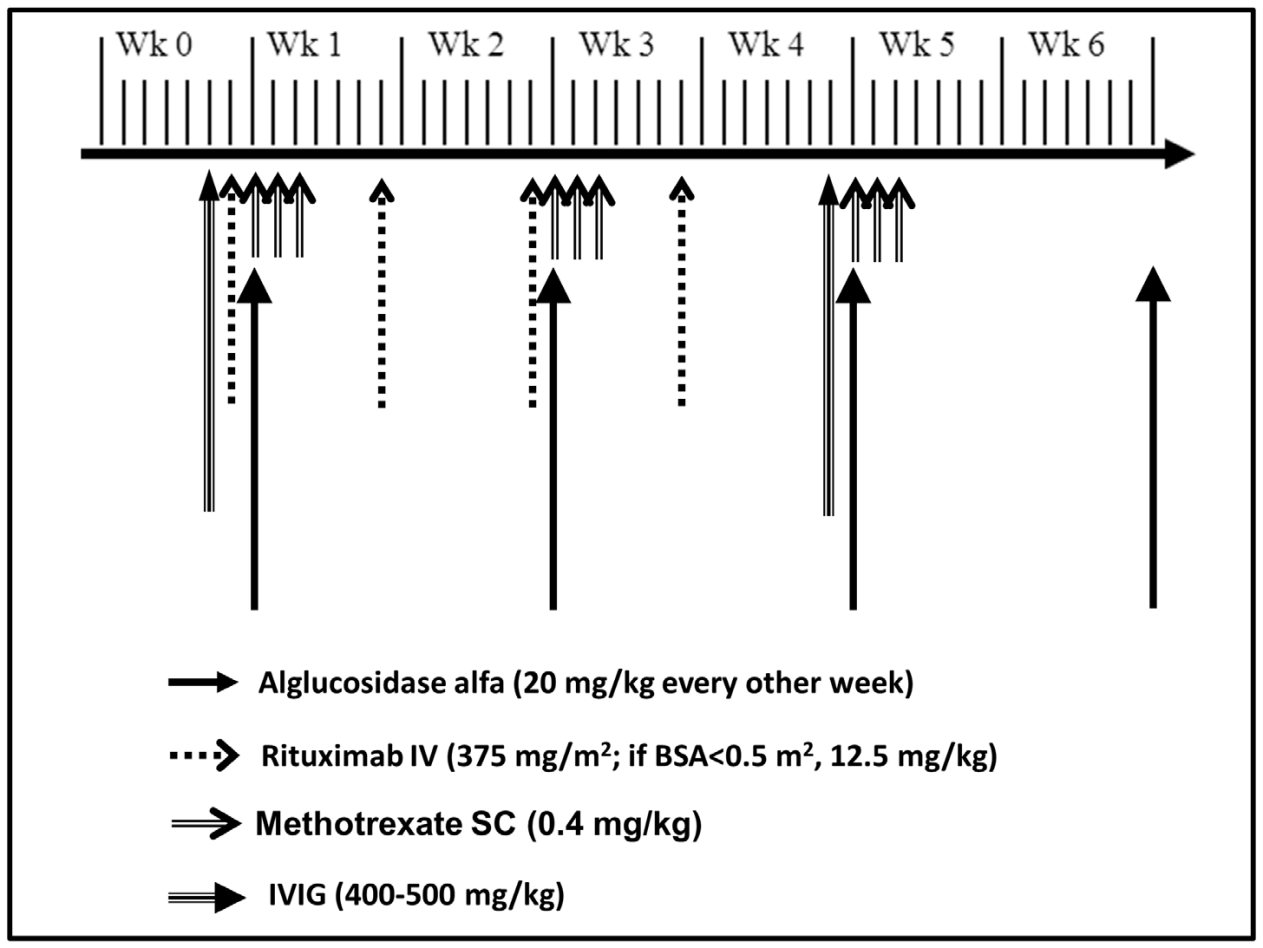
ITI treatment regimen which includes rituximab, methotrexate and intravenous immunoglobulin (IVIG). This short course of ITI regimen (5 weeks) needs to be started together with the first dose of ERT. IVIG is administered on a monthly basis for a period of 5–6 months.

Once CN status was confirmed, the ITI component of this algorithm was implemented along with the initiation of ERT, either as standard of care or after the approval by the respective IRB or ethics committees based on institutional policies. This involved providing all treating physicians with an ITI protocol that included a regimen of rituximab (four weekly doses intravenously) and methotrexate (three doses per week for three weeks subcutaneously), with or without monthly IVIG (Figure 3).

Specific details of the ITI regimen for each patient are shown in Table 1 and are similar to two previously published cases [10]. Based on the algorithm, patients with antibody titers of ≥6,400 at two or more time time-points and CD19 positive B-cell% (CD19%) recovery at ≥5 months were administered another cycle of the same ITI regimen. Patients received alglucosidase alfa (Myozyme®/Lumizyme®) manufactured by Genzyme Corporation (Cambridge, MA) at cumulative doses of 20 mg/kg, administered by infusion every other week based on Myozyme® package insert, or weekly, based on clinical judgment of the treating physicians.

**Table 1:** Details of patient demographics, genotype and immune tolerance induction (ITI) regimen.

	1	2	3	4	5	6	7
**Gender**	Female	Male	Female	Female	Female	Female	Female
**Ethnicity**	Hispanic	African Canadian	Caucasian	African American	African American	Asian	African American
**Allele 1**	c.2608C>T p.Arg870X	c.546+2T>C	c.236_246del p.Pro79ArgfsX13	c.525delT p.Glu176ArgfsX45	c.2560C>T p.Arg854X	c.525_526delTG	c.2560C>T p.Arg854X
**Allele 2**	c.2608C>T p.Arg870X	c.546+2T>C	c.236_246del p.Pro79ArgfsX13	c.2560C>T p.Arg854X	c.2560C>T p.Arg854X	c.525_526delTG	c.2560C>T p.Arg854X
**Age at Diagnosis**	2.5 mo	2.5 mo	2.0 mo	0.3 mo (10 days)	3.0 mo	5.5 mo	3.0 mo
**Age at start of ERT and ITI**	3.0 mo	4.1 mo	2.4 mo	0.4 mo (12 days)	3.5 mo	6.5 mo	4.0 mo
**Time from diagnosis to start of treatment (ERT and ITI)**	0.5 mo	1.6 mo	0.4 mo	0.1 mo (2 days)	0.5 mo	1 mo	1 mo
**ERT (alglucosidase alfa; 20 mg/kg every other week)**	Yes	Yes	Yes	20 mg/kg weekly	Yes	Yes	Yes
**Deviation from actual ITI regimen shown in ** **Figure 3**	IVIG: 1 dose during ITI +2 doses after ITI	Monthly IVIG started at week 4	None	Methotrexate: X 14 weeks (total 42 doses)	None	None	IVIG started at week 4 8 monthly doses +2 extra dose at 8 months
**Repeat ITI**	No	No	No	No	Yes (1 additional cycle at week 35)	Yes (1 additional cycle at week 43)	No
**Length of Myozyme treatment at database lock (in weeks)**	101	92	89	70	59	51	48
**Current Age (as of January 2013)**	127 weeks (29.3 mo)	121 weeks (28.7 mo)	111 weeks (25.8 mo)	84 weeks (19.5 mo)	86 weeks (20 mo)	90 weeks (21.3 mo)	65 weeks (15 mo)*

* Patient 7 died at the age of 15 months (48 weeks into ERT); mo-months; IVIG-intravenous immunoglobulin.

### Clinical Parameters

Baseline and follow-up data pertaining to cardiac, respiratory, motor, and feeding statuses were serially evaluated by healthcare professionals at the respective institutions through October 2012, at which time the database was locked except invasive ventilator free survival which was last evaluated in January 2013. Two-dimensional, M-mode, and Doppler echocardiography were used to assess left ventricular mass index (LVMI).

### Laboratory Parameters

Anti-rhGAA antibodies: Anti-rhGAA IgG antibodies were assessed by Genzyme Corp. (Framingham, MA) at baseline and at regular intervals throughout treatment. Antibody status was ascertained using enzyme-linked immunosorbent assays and confirmed using radioimmunoprecipitation as described previously [5].

### Safety

Safety was assessed by CD19%, frequency of infections, and number of infections requiring hospital admission at or around the time of ITI administration and routine blood tests. Flow cytometry was used to assess CD19%, using standard techniques at each local institution.

### Comparison of CN Patients Treated with ERT+ITI with CN Patients Treated with ERT Monotherapy

We compared invasive ventilator free survival, antibody titers and LVMI values at different time points with the CN ERT+ITI treated patients described here to CN cases treated with ERT monotherapy [8], [9].

### Statistical Analysis

Survival data were analyzed using the Kaplan–Meier method with two-tailed P values generated using the log-rank test [16]. Other reported P values were generated by the Wilcoxon rank sum test for continuous variables. Analyses were performed with STATA version 11.0 (StataCorp LP, College Station, Texas). Because of the limited sample size, all group outcome variable data are presented as medians.

## Results

### Patient Identification and Algorithm for Rapid CRIM Status Determination and ITI Treatment

Seven CN IPD patients were identified at six different institutions in three countries. Through application of this algorithm, CN status was rapidly identified through *GAA* mutation analysis, and subsequently confirmed by western blot analysis of skin fibroblast cells in all seven patients. Demographic information and mutation data for each patient are shown in Table 1. Median age at diagnosis of Pompe disease and CRIM-negative status was 2.5 months (range: 10 days-5.5 months). Due to the ability to predict CN status through mutation analysis alone, there was minimal delay in treatment initiation. Indeed, the delay was primarily due to administrative or regulatory issues (*i.e.*, IRB/ethics committee approval, procurement of resources) rather than to CRIM status determination. The median duration between CN diagnosis and start of treatment was 0.5 months (15 days; range –0.1 months-1.6 months). Median age at start of ITI in conjunction with ERT was 3.5 months (range: 12 days-6.5 months). In all cases, the ITI regimen in Figure 3 was used with minor modifications (Table 1). All patients received alglucosidase alfa (Myozyme®/Lumizyme®) supplied by Genzyme Corporation (Cambridge, MA) at cumulative doses of 20 mg/kg, administered by infusion every other week (n = 6) or every week (n = 1, patient 4) for a median duration of 79 weeks (range: 48–101 weeks).

### Clinical Parameters

**Figure 4:**
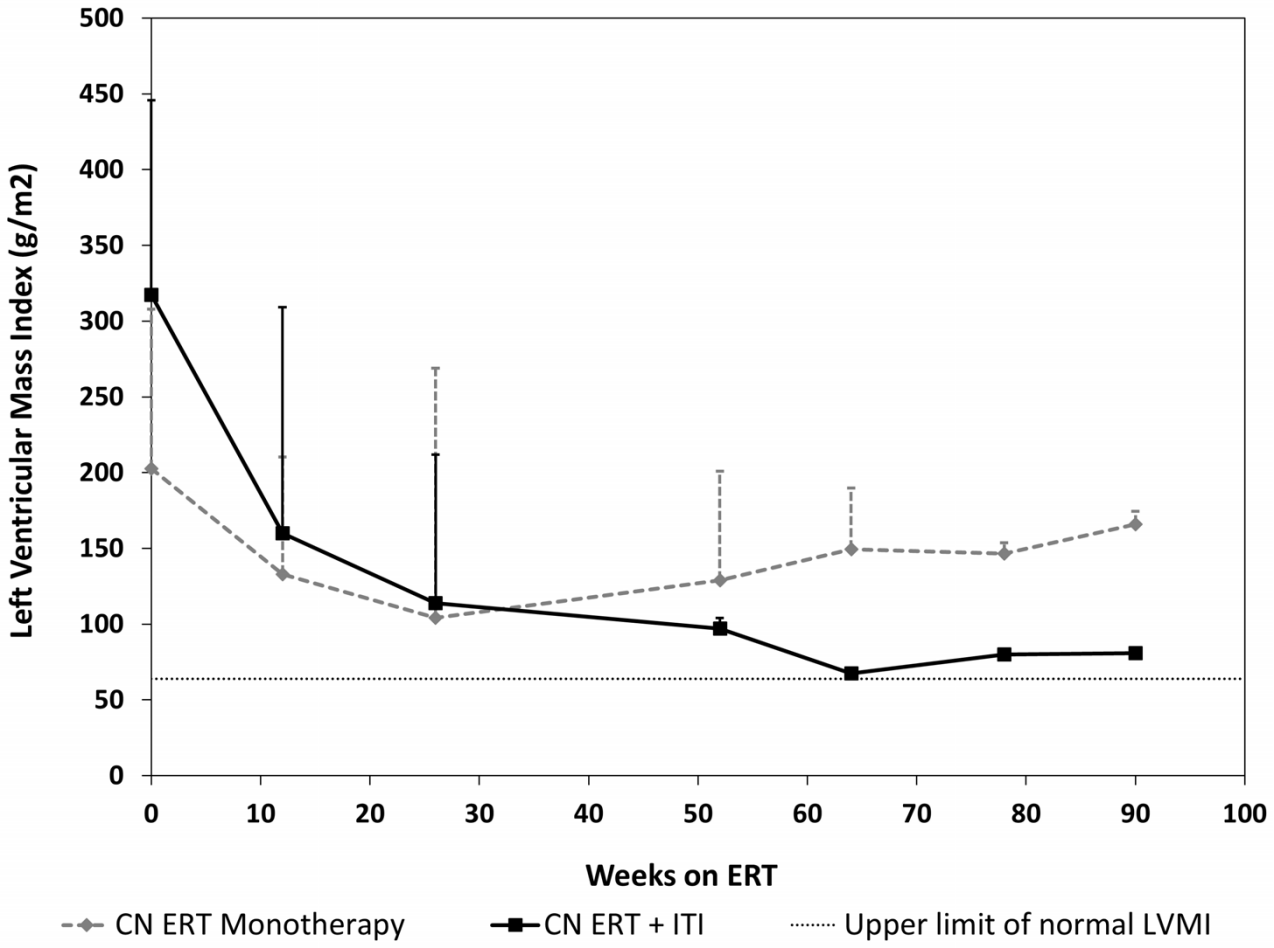
Comparison of median left ventricular mass index (LVMI) values seen over time in CRIM-negative (CN) ERT monotherapy (n = 11) versus CN ERT+ITI (n = 7) treated patients. The upper limit of normal LVMI is 64 g/m^2^ (represented by a horizontal dashed line).

At baseline (prior to start of ERT and ITI regimen; median age: 3.5 months; range: 0.4 months-6.5 months), all patients had increased LVMI (Median: 317 g/m^2^; range: 160–446 g/m^2^) (Table 2, Figure 4). Of the seven patients, three were invasively ventilated, two required supplemental oxygen, one required bi-level positive airway pressure (BiPAP) and one patient required no respiratory support. Clinical parameters over time are shown in Table 2. The median LVMI at the last available time-point was 83 g/m^2^ (range: 64–165 g/m^2^) after treatment with ERT for a median of 75 weeks (range: 36–89 weeks of ERT; n = 7), considerably lower than it was at baseline (median: 317 g/m^2^; range: 160–446 g/m^2^, n = 7) (Figure 4). One of the seven patients (patient 7) required invasive ventilation and subsequently died due to respiratory failure and progressive Pompe disease complications at age 15 months. For the remaining six patients at their most recent assessment, two required no respiratory support, three required BiPAP only at night (of which two required supplemental oxygen), and one patient requiring invasive ventilation at baseline was able to come off of the ventilator for 10–12 hours each day (Table 2).

**Table 2:** Clinical parameters.

		1	2	3	4	5	6	7
**Cardiac Status - LVMI g/m^2^**	**Baseline**	160	446	277	410	317	347	220
	**Current (week)**	81	80	65	92	164	108	83
**Respiratory Status**	**Baseline**	O_2_	O_2_ and BiPAP at night	O_2_	Invasively ventilated	Invasively ventilated	Invasively ventilated	No support required
	**Current**	No respiratory support	O_2_ and BiPAP at night	No respiratory support	Off ventilator 10–12 hours a day	O_2_ and BiPAP at night	BiPAP at night	Invasive ventilation at week 38 ERT until death 2 months later
**Motor Status**	**Baseline**	Head lag and severe hypotonia and motor delay	Head lag, antigravity movements arms>legs,	Severe hypotonia, floppy baby, no head or neck control	Axial hypotonia, withdraws extremities to stimulation, contractures of large joint of upper and lower extremities, weak grasp	Head lag unable to sit or roll over	Severe hypotonia, Antigravity movement in arms	Unable to independently hold head or sit unsupported
	**Current**	Minor head lag when pulled to sit, rolls over, lifts head from prone, sits unsupported, cruises, briefly stands unsupported	Bears weight independently	Ambulates independently	Sits with support; minimal capacity for weight bearing on lower extremities	Standing with support	Marked axial and peripheral hypotonia, yet able to move arms against gravity; near-complete lower extremity immobility	Not able to independently hold head or sit unsupported
**Feeding Status**	**Baseline**	NG tube feeds at age 2 months for dysphagia and fatigue.	NG tube feeds due to aspiration	NG tube feeds started at age 1.4 months	Baseline – G-tube feeds and continues	NJ feeds started at age 3 months	Aspiration and penetration with feeding and started on NG tube feeds at baseline.	NG tube feeds at age 3 months due to aspiration
	**Current**	Oral feeds	GJ tube	Oral feeds	G tube	G tube	G-tube feeds	GJ tube

### Laboratory Parameters

Details on laboratory parameters are shown in Table 3. Four patients (Patients 1, 2, 3 and 4) never seroconverted and remained antibody-free (n = 4; median: 90.5 weeks of ERT; range: 70–101 weeks on ERT) as shown in Table 3. For the remaining three patients (patients 4, 6 and 7), the median peak antibody titer was 1,600 (week 39; patient 7) and 6,400 (weeks 31 and 23 of ERT; patients 5 and 6, respectively) and last available antibody titers were 800 (week 46; patient 7), 3,200 (week 59; patient 5), and 6,400 (week 51; patient 6). Based on the algorithm (Figure 1), two patients with antibody titers of 6,400 (patients 5 and 6) received a second treatment course of the same ITI regimen: one patient (patient 5) had a drop in titer value to 3,200 and the second patient (patient 6) had antibody titers maintained at 6,400 at database lock.

**Table 3:** Laboratory and safety parameters.

		1	2	3	4	5	6	7
**Antibody Titers**	**Baseline**	0	0	0	0	0	0	0
	**Peak (week)**	0	0	0	0	6400 (week 31)	6400 (week 23)	1600 (week 39)
	**Last available data point (week)**	0 (week 101)	0 (week 92)	0 (week 89)	0 (week 70)	3200 (week 59)	6400 (week 51)	800 (week 46)*
**Infections/hospitalization at or around ITI administration**		None	None		None	None	None	None
**Infusion Associated Reactions (IARs)**		One episode; Mild hypotension and rash at 4 weeks ERT	None	None	None	One episode: resolved with pretreatment medication	One episode; resolved with adjusting infusion rate and pretreatment	None
**CD19%**	**Baseline**	Normal	Normal	Normal	Not done	Normal	Normal	Normal
	**CD19% recovery (Weeks on ERT)**	Yes (week 20)	Yes (week 25)	Yes (week 20)	Not done	Increasing CD19% between week 20 and 30	Increasing CD19% between week 20 and 30	Yes (week 31)
	**Last available data point (week)**	Normal (week 46)	Normal (week 25)	Normal (week 75)	Not done	Below normal following second round of ITI	Below normal following second round of ITI	Normal* (week 37)
**Vaccination status (Up-to- date except live vaccines)**		Yes	Yes	Yes	Yes	Yes	Up-to-date till the start of ITI	Yes

* Patient 7 died at age 15 months (48 weeks into ERT).

### Safety

Details on safety related data are shown in Table 3. Only one patient required hospitalization at any time due to infection at or around the time of administration of the ITI regimen: patient 3 developed bronchitis with fever and rash, presumably related to a viral infection, and recovered quickly with no complications. One patient (patient 7) died due to respiratory failure and progressive IPD-related complications that did not appear to be directly related to complications of the ITI protocol. In all cases (n = 6), CD19% dropped to 0 within 2–5 weeks of starting ITI. There was complete recovery of CD19% for the four patients with sufficient follow-up data available (Table 3), three of whom remained anti-rhGAA antibody-free despite CD19 recovery (patients 1, 2 and 3). For one of the patients, CD19% was not measured (patient 4). For the remaining two patients (Patients 5 and 6) there was CD19% recovery along with a small increase in antibody titer, as described earlier, and a second treatment course of the same ITI regimen resulted in the CD19% dropping to 0% (Table 3). There was no decrease in hemoglobin, white cell counts or increase in liver enzymes during the time these patients received ITI.

### Comparison of CN Patients Treated with ERT+ITI with CN Patients Treated with ERT Monotherapy

**Figure 5:**
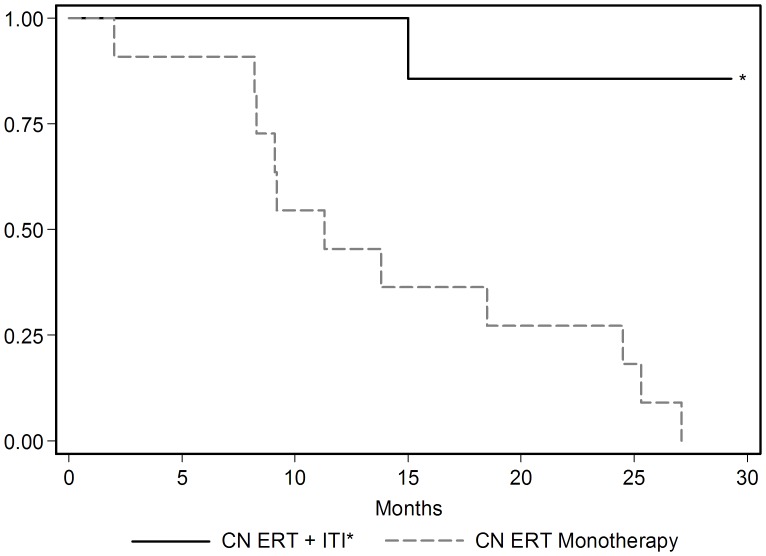
Kaplan-Meier survival curve showing comparison of ventilator-free survival CRIM-negative (CN) ERT monotherapy (n = 11) versus CN ERT+ITI (n = 7) treated patients. *Three patients in the CN ERT+ITI group began the study invasively ventilated, became ventilator-free with treatment, and are currently still alive and ventilator-free. In contrast, all CN patients in ERT monotherapy treated group were invasive ventilator-free at baseline. This observation suggests that in some cases ERT+ITI can even reverse ventilator dependence in CN Pompe patients.

**Figure 6:**
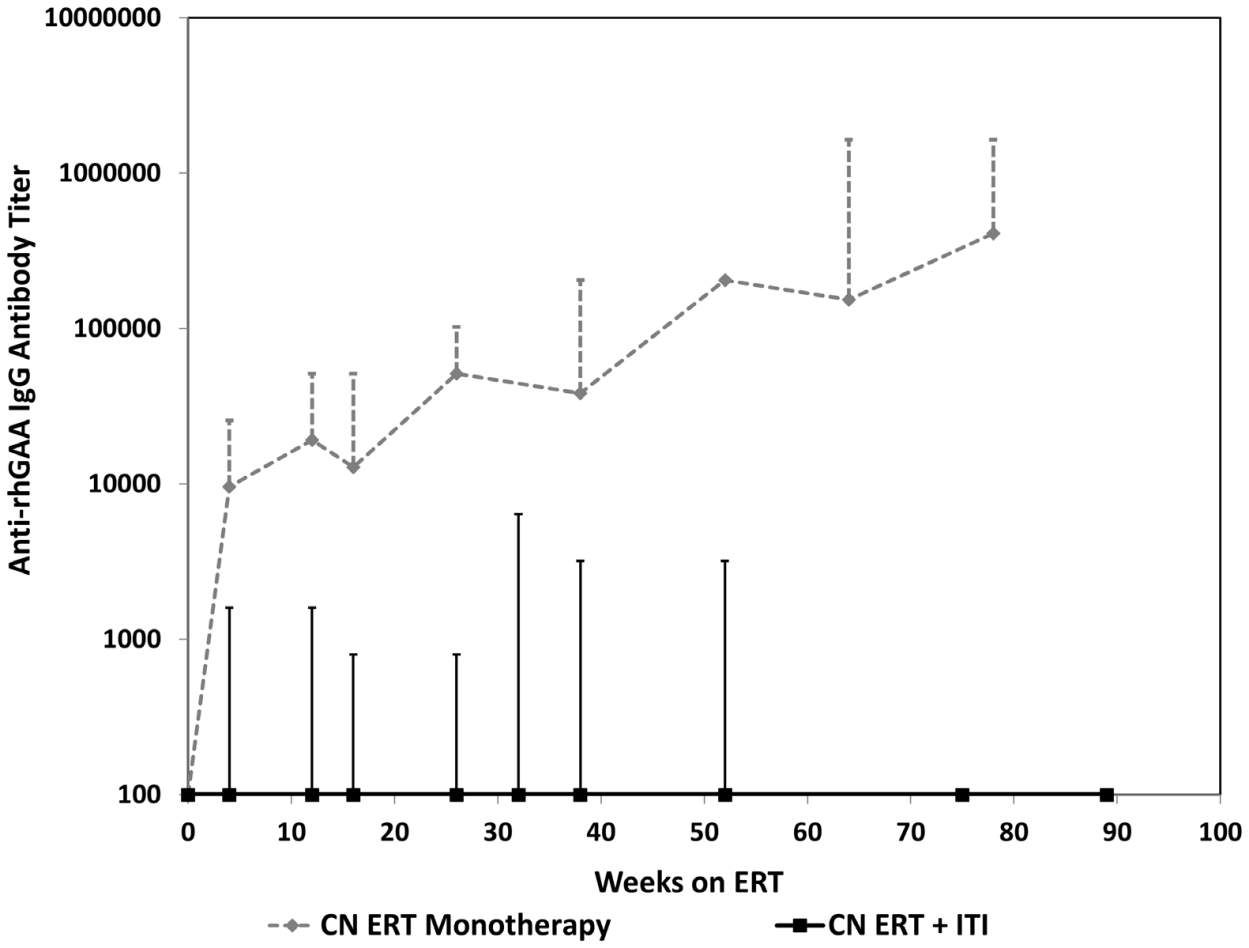
Comparison of anti-rhGAA IgG antibody titers seen over time in CRIM-negative (CN) treated with ERT monotherapy (n = 8) versus CN ERT+ITI (n = 7) treated patients.

All 11 CN patients from a previous study in which they were treated with ERT monotherapy either died or became invasive ventilator dependent by 27.1 months of age [8] versus 1 patient in the ITI+ERT group in this study, who became invasive ventilator dependent and subsequently died at the age of 14.5 months. In terms of invasive ventilator-free survival, there was a statistically significant difference between the CN ERT monotherapy group and CN ERT+ITI group (p = 0.0018; Figure 5). Comparison of antibody titers over time between the two groups shows a clear difference in antibody titers at different time points (Figure 6). None of the CN patients treated with ERT monotherapy were invasively ventilated at baseline, whereas three CN patients treated with ITI+ERT in this study were invasively ventilated at baseline and, remarkably, all were able to come off of invasive ventilation.

When comparing LVMI values over time between the two groups, CN ERT+ITI (n = 7; median: 317 g/m^2^; range: 160–446 g/m^2^) treated group had a higher median LVMI value at baseline (p = 0.02; Figure 4), as compared to CN ERT monotherapy treated group (n = 10; median: 202.58 g/m^2^; range: 89–308 g/m^2^). Both groups had improvement in LVMI for the first 26 weeks [P = 0.26; median LVMI for CN ERT monotherapy treated group (n = 10): 104.15 g/m^2^, range: 56.7–269 g/m^2^; median LVMI for CN ERT+ITI treated group (n = 7): 127 g/m^2^, range: 108–212 g/m^2^], after which there was progression of cardiomyopathy in the CN ERT monotherapy group. In contrast, there was steady improvement in cardiomyopathy for the CN ERT+ ITI treated group (Figure 4). There was a statistically significant difference at week 64 [p = 0.03; Median LVMI for CN ERT monotherapy treated group (n = 3): 149.4 g/m^2^, range: 96.1–189.8 g/m^2^, CN ERT+ITI treated group (n = 4); 75 g/m^2^, range: 65.1–92.7 g/m^2^; Figure 4].

## Discussion

Results of this study show that the algorithm described herein is both feasible and effective for optimizing benefit from ERT by rapidly diagnosing CRIM-negative patients with IPD and starting ITI along with ERT at the earliest possible time point. CN status was determined by mutation analysis alone in all seven cases, with a turnaround time of 48–72 hours. Approximately 90% of all CN patients can be successfully diagnosed by *GAA* mutation analysis alone [15]; in uncertain cases, western blot analysis can be used in conjunction for final CRIM status determination. As more GAA mutations become known, this percentage of cases identified as CN solely by mutation analysis will continue to grow. Moreover, and more importantly, the whole process of CRIM status prediction can be completed in less than a week with proper planning and coordination, as is demonstrated by the cases herein. In our seven cases from around the world, the quick turnaround time of CRIM status determination allowed for start of treatment with ITI plus ERT within 0.5 months (15 days; range: 0.1 months-1.6 months) of CN status determination. Timely introduction of an ITI regimen, such as the one we detailed herein, presents a significant opportunity to further improve the natural history of CN patients with IPD treated with ERT who would otherwise remain at risk of developing HSAT and subsequent clinical decline.

Once a CN patient is identified, the ITI component of the algorithm can be implemented worldwide on an outpatient basis in appropriately-equipped institutions. Findings from this study support a good overall safety and efficacy profile using rituximab, methotrexate and IVIG for ITI in the ERT-naïve setting, despite limited numbers. Five of the seven patients required only a single round of this ITI regimen, while the remaining two patients requiring a second iteration of the same ITI regimen, demonstrated significantly lower antibody titers compared to what has been described in CN patients treated with ERT monotherapy. Not only did titers in our patients remain consistently lower than those usually seen in CN patients on monotherapy, but they were even lower than those seen in so-called low-titer CRIM-positive (LTCP) patients who generally respond well to ERT [9].

While the majority of CN IPD patients develop HSAT, there are rare case reports of CN IPD patients who do not develop HSAT and can have a variable immunological response to ERT [17]–[19]. With multiple factors leading to HSAT formation [9], it is not at this time possible to predict which CN IPD patients will develop HSAT and which will not. Until recently, there have been no known reports of a method to control HSAT once formed. Different combinations of long-term immune modulation with cyclophosphamide, IVIG, plasmapheresis, and increased doses of rhGAA ± rituximab have failed to induce immune tolerance in the setting of an entrenched immune response in CN IPD patients mainly due to inability of those agents/combinations to target antibody-secreting long-lived plasma cells [12], [13]. The only successful attempt at counteracting HSAT utilized a multi-pronged regimen using bortezomib to target plasma cells and rituximab, methotrexate, and IVIG to target naïve and memory B- and T- cells. Yet, the duration and intensity of immune modulation required to counteract the entrenched immune response in the HSAT setting is relatively intense and long [20]. Therefore, rapid prediction of CN status based on GAA mutations and the earliest possible initiation of ERT+ITI is required to optimize long-term clinical outcomes by avoiding cumulative doses of ITI and thus improved safety.

In this treatment algorithm, the ITI regimen included a short course of rituximab, low-dose methotrexate and IVIG. While the exact mechanism by which this regimen induces tolerance is not known, it is believed that the suppression and/or elimination of B- and T-cell populations responsible for antibody formation, with simultaneous up regulation of regulatory T-cells (T_regs_) and/or regulatory B-cells (B_regs_), is important for its success in diminishing the immune response to rhGAA [14]. The ITI regimen described here thus targets immune cells at different levels of the pathway leading to plasma cell formation which is the source of sustained antibody production. The mechanism by which this regimen induces tolerance warrants further investigation using in vitro assays to assess immunologic parameters of tolerance in patient samples and appropriate animal models. It should be noted that all patients, except one (patient 4), were placed on monthly IVIG because of its known immunomodulatory effect [21], and also to provide passive immunity during the period of immune suppression. However, whether IVIG is important in tolerance induction in this setting remains to be investigated.

Since the introduction of ERT for Pompe disease, awareness and early diagnosis have gained importance. Because the therapy is most effective when started early, and methods for dried bloodspot screening for Pompe disease are currently being used to diagnose Pompe disease, newborn screening (NBS) has become a reality in countries such as Taiwan and in several states in the US [22]. In IPD it is vital to commence ERT at the earliest time point possible due to the attendant risks associated with the development of IPD-relevant complications, especially cardiorespiratory failure followed by premature death. The most important and widely accepted goal of newborn screening is to improve health outcomes in the screened population of newborns. Given this goal, screening makes sense only if early detection and treatment will lead to better health outcomes than would be possible if treatment were delayed until the condition became symptomatic or if treatment outcomes are suboptimal despite early start [23]. CN IPD represents the latter category as there is clear evidence that without immune modulation, clinical outcome for CN IPD patients treated with ERT alone is dismal due to formation of HSAT against ERT [8], [9]. While the majority of Pompe cases identified through NBS in Taiwan are CRIM-positive [7], approximately 25.1% of newly diagnosed Pompe cases in US and other parts of the world are CN [15], making a diagnostic and treatment approach which is safe and feasible, like the one described here, even more important.

There are potential limitations specifically related to the ITI regimen reported herein. In particular, this ITI regimen (like most other chemotherapeutic regimens) is relatively non-antigen-specific and has the potential to cause generalized and significant immune suppression. Thus, live vaccines cannot be administered until there is full CD19% recovery and this makes infection with opportunistic pathogens a potential complication that requires close monitoring. Surprisingly, even when administered recurrently over long periods of time [10] very few reports of severe life threatening infections have been reported. This may well be attributable to the use of IVIG which is known to protect against infectious agents in the setting of severe immune suppression while paradoxically acting as an immunomodulatory agent that may enhance immune tolerance [24]. Nonetheless, if severe, life-threatening infections do arise, cessation of ITI could be required and must be balanced against the risks inherently imposed by a CRIM-negative status despite administration of ERT.

Another limitation is the potential need for implementation of more than a single cycle of the regimen, specifically if there is a breakthrough in anti-rhGAA IgG titers. In this study, two of the seven patients required more than one administration of ITI regimen. These patients may have FcR polymorphisms that diminish the efficacy of rituximab [25] or other factors that caused a more rapid progression of the immune response such that long lived plasma cells are mediating the response.

Although the ITI treatment described here prolongs survival and improves significant overall clinical outcomes, closer examination clearly demonstrates residual muscle weakness and gross motor function below age-appropriate levels along with feeding difficulties. However, even in long term CRIM positive survivors, despite long-term treatment with ERT, similar issues are noted [26]. Further follow-up of CN long-term survivors is needed to better understand the overall outcomes of these patients.

The data presented here add to the two previously reported cases of successful use of this ITI regimen in CN patients [10]. Use of the protocol described herein is designed to take place over a relatively brief period of time, and the agents employed have been used extensively for a broad range of conditions, including those in the pediatric population specifically. Moreover, CD19% is associated with the degree of immune suppression and is a useful marker for monitoring the status of immune suppression, but not of immune tolerance. In this study, no patients experienced any major side effects. Moreover, no major illnesses or hospitalizations were directly attributed to the implementation of this protocol, and it was possible to administer vaccines as scheduled upon recovery of CD19 counts. Other approaches to immune tolerance induction, such as non-depleting anti-CD4 mAb, gene therapy, or agents that are antigen specific may prove highly efficacious and avoid prolonged general immune suppression [14]. The algorithm described here will serve as a paradigm going forward. The combination of B- and T-cell targeting agents used in these patients appears to be both safe and efficacious. This regimen, shows promise in altering the natural history of CN IPD patients, and allows for full derivation of the long-term clinical benefits of ERT.
